# Implementation of a first-trimester prognostic model to improve screening for gestational diabetes mellitus

**DOI:** 10.1186/s12884-021-03749-x

**Published:** 2021-04-13

**Authors:** Fieke van Hoorn, Maria P. H. Koster, Anneke Kwee, Floris Groenendaal, Arie Franx, Mireille N. Bekker, On behalf of the RESPECT 2 study group, Anjoke J. M. Huisjes, Marije Lamain-de Ruiter, Eva Stekkinger, Simone A. Vankan-Buitelaar, Mariska A. A. W. Vonk

**Affiliations:** 1 Department of Obstetrics and Gynaecology, University Medical Centre Utrecht, Utrecht University, Lundlaan 6, Utrecht, 3584 EA the Netherlands; 2 Department of Obstetrics and Gynaecology, Erasmus MC, University Medical Centre Rotterdam, Doctor Molewaterplein 40, Rotterdam, 3015 GD the Netherlands; 3 Department of Neonatology, University Medical Centre Utrecht, Utrecht University, Lundlaan 6, Utrecht, 3584 EA the Netherlands

**Keywords:** Prognostic model, Clinical prediction rule, Gestational diabetes mellitus, Selective screening, Implementation, Impact analysis, NoMAD, MIDI, Normalization process theory, Risk communication

## Abstract

**Background:**

Improvement in the accuracy of identifying women who are at risk to develop gestational diabetes mellitus (GDM) is warranted, since timely diagnosis and treatment improves the outcomes of this common pregnancy disorder. Although prognostic models for GDM are externally validated and outperform current risk factor based selective approaches, there is little known about the impact of such models in day-to-day obstetric care.

**Methods:**

A prognostic model was implemented as a directive clinical prediction rule, classifying women as low- or high-risk for GDM, with subsequent distinctive care pathways including selective midpregnancy testing for GDM in high-risk women in a prospective multicenter birth cohort comprising 1073 pregnant women without pre-existing diabetes and 60 obstetric healthcare professionals included in nine independent midwifery practices and three hospitals in the Netherlands (effectiveness-implementation hybrid type 2 study). Model performance (c-statistic) and implementation outcomes (acceptability, adoption, appropriateness, feasibility, fidelity, penetration, sustainability) were evaluated after 6 months by indicators and implementation instruments (NoMAD; MIDI).

**Results:**

The adherence to the prognostic model (c-statistic 0.85 (95%CI 0.81–0.90)) was 95% (*n* = 1021). Healthcare professionals scored 3.7 (IQR 3.3–4.0) on implementation instruments on a 5-point Likert scale. Important facilitators were knowledge, willingness and confidence to use the model, client cooperation and opportunities for reconfiguration. Identified barriers mostly related to operational and organizational issues. Regardless of risk-status, pregnant women appreciated first-trimester information on GDM risk-status and lifestyle advice to achieve risk reduction, respectively 89% (*n* = 556) and 90% (*n* = 564)).

**Conclusions:**

The prognostic model was successfully implemented and well received by healthcare professionals and pregnant women. Prognostic models should be recommended for adoption in guidelines.

**Supplementary Information:**

The online version contains supplementary material available at 10.1186/s12884-021-03749-x.

## Background

Gestational diabetes mellitus (GDM) is a common disorder in pregnancy with an estimated incidence of five to 10 % [1, 2]. GDM is associated with short- and long-term complications affecting both mother and child, such as macrosomia and diabetes mellitus type 2 [3–5]. Timely diagnosis and treatment of GDM improves pregnancy outcomes, therefore, improvement in the accuracy of identifying women who are at risk to develop GDM is warranted [6–9].

Many countries recommend universal testing for GDM by means of an oral glucose tolerance test (OGTT) in the second or third trimester of pregnancy as part of standard obstetric care [1]. However, in low-risk populations testing is often performed selectively in women with one or more risk factors for GDM to prevent many women without GDM being subjected to a burdensome OGTT stressing healthcare budgets and logistics. However, GDM diagnoses could be missed with this strategy [1].

Prognostic models for GDM have the potential improve selective testing [10]. Such models, which weigh and combine readily available predictors, have already been externally validated and outperform current risk factor based selective screening approaches [10–12]. Implementation of a prognostic model has the potential to reduce the number of women unnecessarily undergoing a burdensome oral glucose tolerance test (OGTT) and, consequently, healthcare expenditure. However, at present there is little evidence about the performance, impact and usefulness of prognostic models for GDM in day-to-day obstetric care [13].

The implementation of prognostic models in healthcare is challenging and complex. Contrary to current GDM screening approaches, prognostic models require additional action when predictors have to be entered into the model to generate a woman’s risk [14, 15]. Professionals have to integrate the model into their daily work routine while being influenced by group processes, social conventions, organisational factors and social structures they operate in (normalisation process) [16]. It is important to study outcomes that measure implementation success to advance understanding of implementation processes [17]. Ideally, such an evaluation to assess whether a prognostic model for GDM is feasible for daily clinical use, should be performed before adoption in clinical guidelines [13]. After all, an innovation is not likely to be effective when it is not implemented properly.

Therefore, the aim of this study was to evaluate the implementation of an externally validated prognostic model to improve selective screening for GDM through the assessment of implementation outcomes and model performance involving both obstetric healthcare professionals (OHP) and pregnant women. The identified barriers and facilitators may also provide insights into enhancing implementation of healthcare innovations in other settings.

## Methods

### Design and study population

We performed an effectiveness-implementation hybrid type 2 study in a prospective multicentre regional birth cohort to evaluate both implementation outcomes and performance of a first-trimester prognostic model for GDM. The study was performed between December 2016 to January 2018 in nine midwifery practices and three hospitals in the central region of the Netherlands (Risk EStimation for PrEgnancy Complications to provide Tailored care study part two (RESPECT2): implementation of a prognostic model for GDM).

The implementation was evaluated among OHP from the participating centres. All obstetric healthcare professionals, including midwives working in independent midwifery practices (primary care) or clinical midwives, residents in obstetrics and obstetricians employed in hospitals (secondary or tertiary care) who used the model were asked to participate. All pregnant women in the participating centres received the replaced standard care (i.e. the prognostic model for GDM). Pregnant women ≥18 years old, were asked to provide informed consent for use of medical data for research purposes and to fill out questionnaires. Exclusion criteria were < 18 years old, inability to provide informed consent or to respond to online questionnaires in Dutch. The RESPECT2 study was approved by the medical ethics committee of the University Medical Centre Utrecht on 15 November 2016 (protocol 16/741).

### Selective screening for GDM by the prognostic model

All participating centres replaced their current screening approach in which testing is performed selectively in women with one or more risk factors for GDM [14], by the prognostic model for GDM as standard care during the study. The model contained the following predictors: maternal age, body mass index (BMI), ethnicity, first-degree relative with any type of diabetes mellitus, history of GDM and first-trimester venous glucose. The full equation plus information on threshold consensus is shown in Box S1 in Additional file 1. An implementation plan was set up using the refined compilation of implementation strategies as defined by Powell et al. 2015 (Box S2 in Additional file 1) [18].

The prognostic model was applied by OHP through a secured online data-collection platform (Research Online©). The result was displayed as either low- or high risk with subsequent directive recommendations for care. A two-page leaflet to take home was discussed with high-risk women. This included general information on GDM, risk of GDM, preventive measures and their care pathway. The care pathway included testing for GDM at 24–28 weeks of gestation with an OGTT and an ultrasound examination to check for macrosomia or polyhydramnios at 30–32 weeks of gestation. In the part ‘What can I do?’, healthy gestational weight gain, exercise (at least 30 min a day) and healthy nutrition with referral to the website of Netherlands Nutrition Centre for pregnant women was advised. Also, consultation with a dietician was suggested. Low-risk women only received information on their risk for GDM. Note that all pregnant women, regardless of their GDM risk-status, underwent an OGTT when they developed symptoms of GDM at any point in pregnancy. See Box S3 in Additional file 1 for the diagnosis and treatment of GDM.

### Implementation outcomes

Implementation outcomes as defined by Proctor et al. (adoption, acceptability, appropriateness, feasibility, fidelity, penetration, sustainability) were assessed 6 months after implementation using the validated NoMAD and MIDI instruments plus additional questions and indicators [17, 19–22]. The NoMAD is based on the Normalization Process Theory, which states that normalization is a process of embedding and integrating healthcare innovations in routine care as a product of action of individuals and groups [20]. The four constructs in this theoretic framework are: coherence, cognitive participation, collective action and reflexive monitoring [19, 20]. The MIDI is developed to identify factors influencing the use of an implemented intervention comprising of four constructs measuring determinants associated with the innovation, user, organisation and socio-political context [22].

In Table 1 it is shown how the implementation outcomes were defined in the context of our study and through which items they were assessed. The full subconstructs and details about scaling of the survey administered to OHP are provided in Table S1 in Additional file 1.

**Table 1:** Definition of implementation outcomes and their assessment

Outcome	Definition	Indicator	OHP survey item^a^
Adoption	The initial decision to implement the prognostic model	The number of centres that started with the implementation of the prognostic model, divided by the total number of centres agreed to participate.	CP3
Acceptability	The perception among obstetric healthcare professionals that the prognostic model is agreeable, palatable, or satisfactory	*NA*	I1, I2, I4, U8, U17, C1, C3, C4, RM1–3
Appropriateness	The perceived fit, relevance, or compatibility of the prognostic model for a) midwifery practices, hospitals, obstetric healthcare professionals, pregnant women, or b) to improve selective screening for GDM	The number of pregnant women who appreciated information about their risk for GDM and how to decrease it, divided by the total number of women who responded to the questionnaire^b^	I7, U9, U10–12, GN1, C2, CP2, CA3
Feasibility	The extent to which the prognostic model can be successfully used or carried out within the midwifery practice or hospital	*NA*	I5, U13, U16, O19, O21, O23–27, GN2, CP1, CA1, CA2, CA4–7, RM5
Fidelity	The degree to which the prognostic model was implemented as it was described in the original protocol	*Fidelity: t*he number of pregnant women who received the correct care pathway, divided by the total number of women in the study population.^c^ *Safety:* the number of women with GDM that were selectively tested for GDM, divided by the total number of women with GDM.^c^ *Efficiency:* the number of women without GDM that were not selectively tested for GDM, divided by the total number of women without GDM.^c^	*NA*
Penetration	The integration of the prognostic model in the midwifery practices and hospitals	The number of pregnant women for whom the prognostic models was filled out, divided by all pregnant women.^c^ The number of pregnant women who resported to have received information about their risk for GDM, divided by the total number of women who responded to the questionnaire^b^	U14, U15
Sustainability	The extent to which the use of the prognostic model is maintained within a midwifery practice or hospital	The number of centres that continued using the prognostic model after the study period, divided by the total number of centres that participated in the study^d^	I6, O20, O28, GN3, CP4, RM4, E1–5.

*OHP* Obstetric healthcare professional, *GDM* Gestational diabetes mellitus, *NoMAD* Normalization MeAsure Development Instrument [19–21], *MIDI* Measurement Instrument for Determinants of Innovations instrument [22], *NA* Not applicable

^a^Full items are provided in supporting table 1

^b^Numbers derived from the questionnaire send to pregnant women

^c^Extracted from the standardised case report forms and displayed in Fig. 3

^d^Centres were asked by e-mail whether they had continued using the prognostic model after the study period

### Data collection

OHP received a survey 6 months after implementation (reminders after 3, 7 and 10 days). Collected demographics included age in years, sex, profession (midwife employed in an independent midwifery practice, midwife employed in a hospital, resident in obstetrics, obstetricians), and years of OHP experience.

Characteristics of pregnant women were documented by OHP via the model and pregnancy outcomes were extracted from medical records by the research team in a standardised case report form (Box S4 in Additional file 1). Pregnant women received a questionnaire at 22 weeks of gestation and 4 weeks after their estimated date of delivery including questions and statements on risk communication and lifestyle in relation to pregnancy outcome (reminders after seven and 14 days).

### Statistical analysis

Women with a multiple pregnancy, fetal demise < 16 weeks of gestation or who were lost to follow-up were excluded for analysis. Characteristic and outcomes of pregnant women were assessed for the overall study population as well as stratified for risk (high-risk versus low-risk) and GDM diagnosis. A responder versus non-responder analysis was performed for both OHP and pregnant women; a pregnant woman was defined as a responder when she filled out one or both questionnaire(s).

Model performance was assessed by c-statistics and calibration-plots. The sensitivity and specificity of the prognostic model were compared to the formerly used selective screening approach [14]. A reclassification plot illustrated how the proportions of women who were true positive, true negative, false positive or false negative for GDM altered by using the prognostic model compared to the former approach.

The subconstructs of the NoMAD and MIDI instruments were scored on a 5-point Likert scale from 0 (strongly disagree) to 5 (strongly agree). Subconstructs to which ≥20% of the healthcare professionals responded with ‘strongly disagree’ or ‘disagree’ were considered barriers, and those to which ≥80% responded ‘strongly agree’ or ‘agree’ were considered facilitators [23]. A single composite score for a subconstruct consisting of multiple statements, total scores for the instruments and the combined total score were calculated by averaging the statements, the scores of all constructs and the total scores of both instruments, respectively. Internal consistency of the constructs and total scores was assessed with Cronbach’s Alpha for all subconstructs and when the least coherent subconstruct was dropped (α: < 0.50 ‘unacceptable’, 0.50–0.59 ‘poor’, 0.60–0.69 ‘questionable’, 0.70–0.79 ‘acceptable’, 0.80–0.89 ‘good’, ≥0.90 ‘excellent’).

Analyses were performed in SPSS statistics version 25 (SPPS Inc., Chicago, IL) and R version 3.5.1 for windows using ‘rms’ and ‘calibrate’ packages (http://cran.r-project.org).

## Results

### Study population

The majority of the 60 OHP was female (*n* = 58; 97%) and worked as midwifes in primary care (*n* = 45; 75%) (Table 2). The number of responders to the survey 6 months after implementation was 42 (70%). No significant differences in demographics were observed between responders and non-responders.

**Table 2:** Determinants of healthcare professionals employed at the implementation sites overall and stratified by responder type

Determinant	Overall (***n*** = 60)	Responder (***n*** = 42)	Non-responder (***n*** = 18)	*P-value*
Age (years)	40 (31–51)^a^	40 (32–52)^b^	34 (30–43)^c^	*0.365*
Sex (female)	58 (96.7)	41 (97.6)	17 (94.4)	*0.530*
Profession				
Midwife, employed in independent midwifery practice^d^	45 (75.0)	32 (76.2)	13 (72.2)	*0.145*^¶^
Midwife, employed in hospital^e^	3 (5.0)	1 (2.4)	2 (11.1)	
Resident in obstetrics ^e^	3 (5.0)	1 (2.4)	2 (11.1)	
Obstetricians^d^	9 (15.0)	8 (19.0)	1 (5.6)	
Employed in obstetrics (years)	12 (8–23)^a^	12 (8–24)^b^	10 (4–18)^c^	*0.350*

Values are median (interquartile range) or number percentage

^¶^*P*-value when comparing all four categories (primary care compared to secondary/tertiary care (*p* = 0.745); midwifes compared to doctors (*p* = 0.673))

^a^Missing for three respondents

^b^Missing for one respondent

^c^Missing for two respondents

^d^Primary care

^e^Secondary or tertiary care

A total of 1940 pregnant women were assessed for eligibility of whom 798 met one of the exclusion criteria or did not provide informed consent. Another 69 women were excluded because they had a multiple pregnancy (*n* = 27), fetal demise < 16 weeks of gestation (*n* = 11) or were lost to follow-up (*n* = 31), leaving 1073 women for analysis. The age and BMI of the study population were 31.4 ± 4.3 years and 23.7 (IQR 21.4–27.3) kg/m^2^, respectively. The prognostic model classified 352 women (32.8%) as high-risk for GDM. Gestational diabetes mellitus was diagnosed in 81 women (7.5%) of whom 71 (87.7%) were classified as high-risk. Characteristics and outcomes of the study population overall and stratified for risk for and diagnosis of GDM are described in Table 3. Rates of high-risk status for and diagnosis of GDM were similar between the 672 responders (62.6%) and 401 non-responders (37.4%) to the questionnaires, however, non-responders were slightly younger and more often of non-Caucasian ethnicity (Table S2 in Additional file 1).

**Table 3:** Characteristics of the study population overall, and stratified for risk for and diagnosis of gestational diabetes mellitus

Characteristics	Low risk (*n* = 721)		High risk (*n* = 352)		***Overall*** *(n = 1073)*
	no GDM (*n* = 711)	GDM (*n* = 10)	no GDM (*n* = 281)	GDM (*n* = 71)	
Age (years)	31.0 (4.2)	30.1 (3.0)	32.3 (4.4)	32.2 (4.7)	*31.4 (4.3)*
Body mass index (kg/m^2^)	22.5 (20.6–24.3)	23.6 (22.0–24.8)	27.8 (25.0–31.2)	30.1 (27.8–33.3)	23.7 (21.4–27.3)
Ethnicity (Caucasian)	641 (90.2)	9 (90.0)	203 (72.5)	51 (71.8)	*904 (84.2)*
Parity (nulliparous)	318 (44.7)	5 (50.0)	93 (33.1)	24 (33.8)	*440 (41.0)*
Spontaneous conception	668 (94.0)	10 (100)	256 (91.1)	69 (97.2)	*1003 (93.5)*
Pre-existent hypertension	4 (0.6)	0 (0.0)	12 (4.3)	2 (2.9)	*18 (1.7)*
Polycystic ovarian syndrome	14 (2.0)	0 (0.0)	6 (2.1)	5 (7.0)	*24 (2.2)*
History of gestational diabetes mellitus	0 (0.0)	0 (0.0)	14 (5.0)	17 (23.9)	*31 (2.9)*
History of macrosomia^a^	19 (2.7)	1 (10.0)	12 (4.3)	7 (9.9)	*39 (3.6)*
History of unexplained intra-uterine fetal demise	1 (0.1)	0 (0.0)	1 (0.4)	2 (2.8)	*4 (0.4)*
Family history of diabetes^b^	39 (5.5)	1 (10.0)	124 (44.1)	27 (38.0)	*190 (17.7)*
First trimester glucose (mmol/L)	4.6 (4.3–4.9)	4.5 (4.1–4.8)	5.0 (4.7–5.4)	5.3 (4.9–5.6)	4.7 (4.4–5.1)
Hypertensive disorders of pregnancy	73 (10.3)	2 (20.0)	43 (15.3)	16 (22.5)	*134 (12.5)*
Induction of birth	139 (19.5)	5 (50.0)	75 (26.7)	37 (52.1)	*256 (23.9)*
Mode of birth (spontaneous)	573 (80.6)	3 (30.0)	217 (77.2)	47 (66.2)	*840 (84.4)*
Postpartum hemorrhage > 1000 ml	60 (8.4)	0 (0.0)	19 (6.8)	8 (11.3)	*87 (8.1)*
Maternal death	0 (0.0)	0 (0.0)	0 (0.0)	0 (0.0)	*0 (0.0)*
Gestational age at birth (days)	281 (274–286)	276 (270–279)	280 (272–287)	273 (266–281)	280 (273–286)
Birthweight (grams)	3500 (3140–3810)	4008 (3712–4357)	3507 (3155–3850)	3480 (3088–3958)	3500 (3140–3840)
Small-for-gestational-age^c^	83 (11.7)	0 (0.0)	35 (12.5)	6 (8.5)	*124 (11.6)*
Large-for-gestational-age^a^	55 (7.8)	6 (60.0)	39 (13.9)	15 (21.1)	*118 (11.0)*
Apgar-score < 7 after 5 min	11 (1.6)	1 (10.0)	6 (2.2)	1 (1.4)	*19 (1.8)*
Shoulder dystocia	18 (2.5)	1 (10.0)	10 (3.6)	1 (1.4)	*30 (2.8)*
Birth trauma	5 (0.7)	0 (0.0)	0 (0.0)	1 (1.4)	*6 (0.6)*
Hypoglycemia < 2.6 mmol/L	56 (7.9)	3 (30.0)	28 (10.0)	20 (28.2)	*107 (10.0)*
Neonatal intensive care admission	20 (2.8)	0 (0.0)	9 (3.2)	2 (2.8)	*31 (2.9)*
Perinatal death > 22 weeks gestational age	3 (0.4)	0 (0.0)	3 (1.1)	0 (0.0)	*6 (0.6)*

Values are mean (SD), median (interquartile range) or number (percentage)

*GDM* Gestational diabetes mellitus, *Low risk* Low risk for GDM, *High risk* High risk for GDM. Predictors in prognostic model: age, body mass index, ethnicity, first trimester glucose level, family history of diabetes, GDM in a previous pregnancy

^a^Birthweight percentile > 90 [24]

^b^First degree family member with any type of diabetes mellitus

^c^Birthweight percentile < 10 [24]

### Model performance

**Fig. 1:**
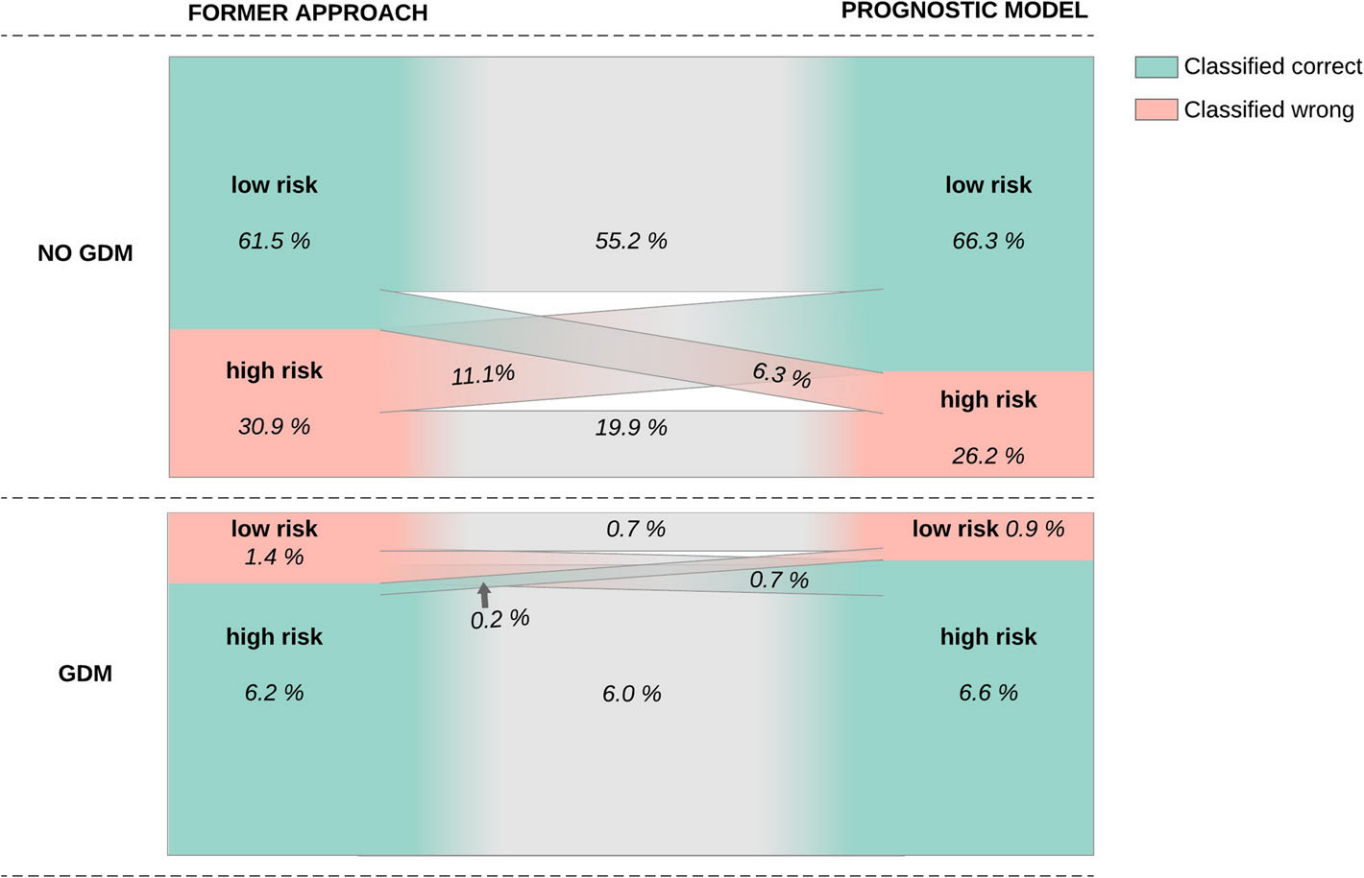
Reclassification plot of the former approach for selective screening for gestational diabetes mellitus compared to a prognostic model

The prognostic model showed good discrimination, illustrated by a c-statistic of 0.85 (95%CI 0.81–0.90), and adequate calibration (Figure S1). The prognostic model classified 4.3% fewer women as high-risk for GDM (32.8% compared to 37.1%) and classified 5.3% more women correctly than the former approach (Fig. 1). Moreover, the prognostic model outperformed the former approach on all test characteristics illustrated by a sensitivity and specificity of 87.7 (95%CI 30.0–35.7) and 71.7 (95%CI 68.8–74.5) compared to 81.5 (95%CI 71.3–89.2) and 66.5 (95%CI 63.5–69.5).

### Implementation outcomes

OHP were predominantly positive about the model with an overall score on implementation outcomes of 3.7 (IQR 3.3–4.0) (Table S3 in Additional file 1). Scores were not associated with the respondent’s age (*p* = 0.735*)*, sex (*p = 0.571),* profession (*p* = 0.736), or number of years employed in obstetrics (*p* = 0.862). The internal consistency of the MIDI and NoMAD total scores were good to excellent (0.86 ≤ alpha≤91 and 0.85 ≤ alpha≤0.88, respectively) (Table S3 in Additional file 1).

### Adoption

**Fig. 2:**
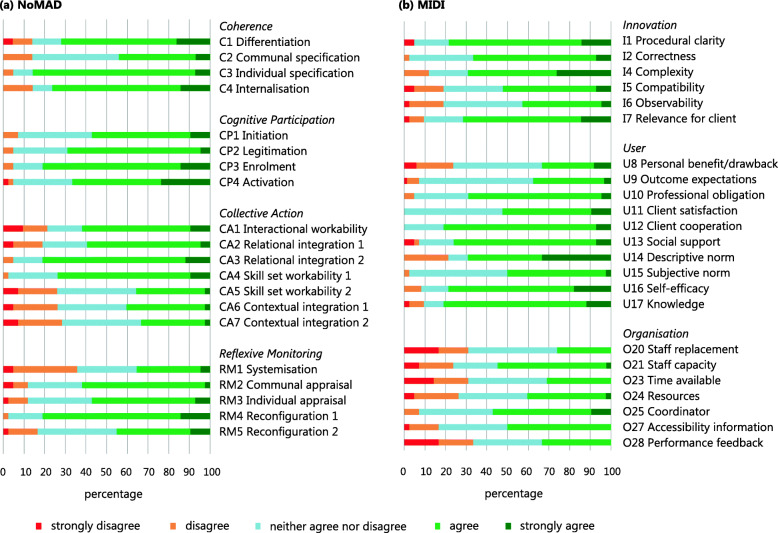
Distribution of responses of obstetric healthcare professionals on NoMAD (**a**) and MIDI (**b**) subcontructs 6 months after the implementation of a prognostic model for gestational diabetes mellitus

All centres that made the initial decision to adopt the prognostic model for GDM also started the implementation process. On an individual level, the majority (*n* = 34; 81%) of OHP was open to start working with the model (Fig. 2; CP3).

### Acceptability

Most OHP scored positive on the procedural clarity of the prognostic model, adequate knowledge to use it and on how the model affects the nature of their work (*n* = 33; 79%, n = 34; 81%, *n* = 36; 86%, respectively) (Fig. 2; I1, U17, C3). However, some reported no awareness of scientific reports about the effects of the innovation (*n* = 15; 36%), and reported no improvement client relationships as a personal drawback (*n* = 10; 24%) (Fig. 2; RM1; U8).

### Appropriateness

OHP scored neutral to positive on all subconstructs concerning their perceived fit, relevance or compatability of the prognostic model. They found the prognostic model relevant for their clients (*n* = 30; 71%) and had confidence in their colleagues’ ability to use the model (*n* = 34; 81%) (Fig. 2; I7, CA3). OHP expected that high-risk women would accept testing on GDM (*n* = 38; 91%), whereas they scored neutral on the outcome expectations regarding effects of lifestyle advice (Fig. 2; U9). OHP scored a 7.5 (IQR 6.5–8.1), on a scale from 0 = ‘not al all’ to 10 = ‘completely’, on the question “When you use the prognostic model, how familiar does it feel?” (Table S3 in Additional file 1; past normality).

Pregnant women appreciated first-trimester information on GDM risk-status and lifestyle advice to achieve risk reduction, in both the second trimester (*n* = 556; 89% and *n* = 564; 90%) and postpartum (*n* = 437; 95% and *n* = 433; 94%). These results were similar for low- and high-risk women*.*

### Feasibility

OHP ability to discuss lifestyle advice with high-risk women was identified as a facilitator (*n* = 36; 86%). They also scored positive on self-efficacy to apply the prognostic model as intended (*n* = 33; 79%), and agreed that they could count on adequate assistance from colleagues when needed (*n* = 32; 76%) (Fig. 2; U13, U16). OHP scored a 7.0 (IQR 5.1–8.2), on a scale from 0 = ‘not al all’ to 10 = ‘completely’, on the question “Do you feel that the prognostic model is currently a normal part of your work?” (Table S3 in Additional file 1; current normality). Barriers included lack of staff capacity (*n* = 10; 24%), time available (*n* = 13; 31%), training (*n* = 11; 26%), resources (*n* = 11; 26%), support (*n* = 12; 29%) and formal ratification by the management (*n* = 27; 64%), compatibility to (*n* = 8; 19%) and easy integration (*n* = 9; 21%) into their existing work, as well as the presence of concurrent innovations (*n* = 37; 88%) (Fig. 2; O21, O23, CA5, CA6, CA7, O19, I5, CA1, O26).

### Fidelity

**Fig. 3:**
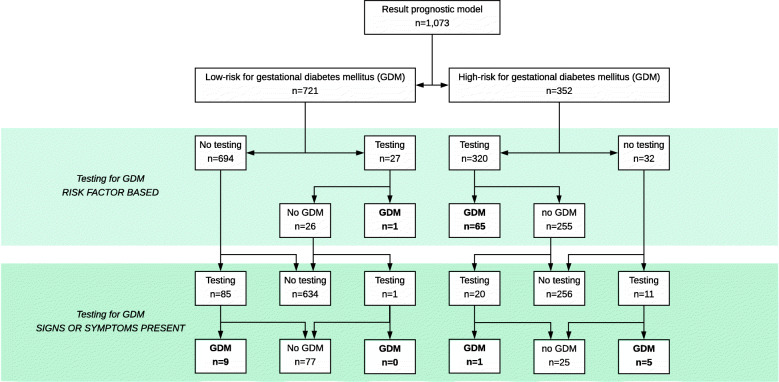
Flowchart of testing for gestational diabetes mellitus after implementation of a prognostic model

Testing for GDM by the prognostic model was performed as intended in 1021 women (95%) (Fig. 3).

In the low-risk group, 694 (96%) women received care conforming to protocol. In 24 (3%) low-risk women where the advice of the prognostic model was violated, the former selective testing approach for GDM was applied. Other reasons were: hyperglycaemia-inducing medication (*n* = 1), wrong risk-status assigned due to administrative error (*n* = 1), or unknown (*n* = 1).

In the high-risk group, 327 (93%) women received care conforming to protocol. The reasons for the 25 (7%) protocol violations were: testing refused by pregnant women (*n* = 3), not advised by OHP (*n* = 3), wrong risk-status assigned due to administrative error (*n* = 7), or unknown (*n* = 12). Withdrawal for risk factor based testing for GDM was not considered as a protocol violation in seven high-risk women, because they had a preterm birth (*n* = 1) or already received an OGTT because of symptoms (*n* = 6) before testing was planned.

The impact of the prognostic model in clinical practice, combining model performance and fidelity, resulted in 84.0% safety (*n* = 68/81) and 70.3% efficiency (*n* = 697/992).

### Penetration

The prognostic model was filled out for 1038 (97%) women. OHP thought that most of their direct colleagues applied the model as intended and also appointed them as the most important influencers on whether or not to apply the prognostic model (Fig. 2; U15; U14).

Low-risk women reported less often that they were informed about their risk for GDM compared to high-risk women, 269 (62%) and 168 (86%) women, respectively. From the women who did receive information, 350 (80%) women remembered their risk correctly, 36 (8%) wrong and 51 (12%) did not remember their risk for GDM; these percentages were not different for low-risk compared to high-risk women (*p* = 0.199).

### Sustainability

A total of 27 (64%) OHP would like to continue using the prognostic model; ten (24%) of them responded neutral. Eight (19%) OHP did not find the effect of using the model clearly observable, 13 (31%) of them reported negative on replacement when staff leaves and 14 (33%) on performance feedback (Fig. 2; I6, O22, O28). With regard to the prognostic model becoming a normal part of their work in the future, OHP scored an 8.0 (IQR 7.0–9.0) on a scale from 0 (not at all) to 10 (completely) (Table S3 in Additional file 1; future normality). They preferred that the model would be integrated in the electronic patient file (*n* = 36; 85%) and felt that feedback can be used to improve the prognostic model (*n* = 34; 81%).

## Discussion

### Main findings

In a prospective regional cohort of 1073 pregnant women, we showed that a first-trimester prognostic model was successfully implemented as a directive clinical prediction rule, classifying women as low- or high-risk for GDM with distinctive care pathways. This was illustrated by the excellent fidelity, good model performance and impact on selective screening for GDM. Moreover, the application of the model and the subsequent care pathways were well received by OHP and pregnant women.

### Strengths and limitations

The major strength of this study is the multifaceted approach, since it is advocated that the impact of prognostic models should be evaluated as complex interventions because the introduction of a model with subsequent management recommendations consists of multiple interacting factors (i.e. model performance, implementations outcomes, stakeholders’ perspectives) [25, 26]. Furthermore, comprehensive evaluation of implementation outcomes in studies introducing a prognostic model are scarce [26], especially using validated quantitative NoMAD and MIDI instruments. Additionally, our prospective study of a large multicentre cohort of pregnant women recruited in all types of obstetric care facilities with little missing data of pregnancy outcomes had the preferable design to measure the implementation process [19, 22].

The inclusion of a control group would have been informative with regard to assessing improvements in medical outcomes, i.e. a step-wedged cluster randomized controlled trial, however, such a design is far more time-consuming and does often not reflect ‘real’ clinical practice [26]. The use of online surveys enabled us to reach many OHP and pregnant women, although combining this data with qualitative deepening of the reasons behind responses could have provided even more insights into factors influencing the implementation process. Also, subjects with a positive attitude towards the model may have been more eager to respond, and may thus be overrepresented. Although response rates were acceptable and no major differences in characteristics were found between responders and non-responders this could, to some extent, have led to a non-response bias. The primary aim of this study was to evaluate the implementation of a prognostic model to improve selective testing for GDM, therefore it was not possible to apply universal testing at the same time. This may have resulted in some missed GDM cases in the low-risk group. We assume that this concerns a small proportion, since women who developed symptoms of GDM at any point in pregnancy did receive an OGTT. Nevertheless, these false-negatives might have led to overestimation of model performance. Although a formal combined diet and exercise intervention was not part of the care pathway of high-risk women, it is possible that the included lifestyle advice may have decreased the GDM incidence in this group to some extent, which might have led to underestimation of model performance.

### Interpretation

This is one of few studies evaluating the introduction of a prognostic model in obstetric care [13]. By our knowledge there has only been one previous study that evaluated the impact of prediction tools for several adverse pregnancy outcomes, including GDM, on perinatal outcomes and costs in a before-after study [27]. This study found a significant reduction of a composite of perinatal outcomes in nulliparous women and was cost-effective [27]. However, it is unknown to what degree the prognostic model for GDM contributed to these results and neither implementation outcomes nor stakeholders’ perspectives were evaluated.

Our study identified several facilitators for the implementation of a prognostic model for GDM in routine obstetric care. We found that the field was ready for its introduction and that OHP had confidence in client cooperation, positive outcome expectations, enough knowledge and understanding how the model affected their work. Most importantly, OHP had confidence in their colleagues’ and their own ability to apply the model for every woman and to discuss lifestyle advice with high-risk women. This is important since self-efficacy has been found to be the strongest predictor for completeness of use [22, 28, 29]. In case of non-adherence, we identified similar reasons as in a recent review on impact analysis studies of clinical prediction rules, including: fear of missing the diagnosis, preference for own clinical judgement and patient request [30]. Finally, it is encouraging that OHP think that feedback about the model and corresponding care pathways can be used to improve these, as this is a commonly reported facilitator that could enhance further integration and maintenance in clinical practice [21, 31–35].

Despite scores on self-efficacy, which were high, a number of OHP did not find it easy to integrate the model into their routine and reported lack of training and awareness of reports about model effectiveness. In contrast to personal knowledge and skills, the other barriers concerned external factors regarding organisation or management, such as insufficient time, staff capacity, management support, formal ratification and resources. Nevertheless, these impeding factors might comprise more than only the implementation of the prognostic model, since 88% of OHP reported that other changes in the organisation affected the implementation with the subsequent stress and heavy workload hindering implementation [36]. The identified themes are in line with previously identified barriers regarding the use of clinical prediction rules in practice [26]. Most barriers were found in the constructs ‘collective action’ (NoMAD) and ‘organisation’ (MIDI) concerning feasibility. Moreover, lack of formal ratification by management was found to be strongly associated with unsuccessful implementation in previous studies [22, 29]. These findings may indicate that the implementation strategy was predominantly a bottom-up approach and that improvements can be made with more emphasis on how OHP work together within an organisation to enact the model in their daily routine and by more comprehensive involvement of the management [20, 21].

The performance of the implemented model in our study was higher than previous development, external validation and update studies with a c-statistic of 0.85 (95%CI 0.81–0.90) compared to c-statistics ranging from 0.70 (95%CI 0.68–0.73) to 0.80 (95%CI 0.76–0.84) [10–12, 37]. In clinical practice, this resulted in less pregnant women requiring testing for GDM while more GDM cases were timely identified, which may reduce perinatal morbidity and subsequent healthcare expenditure [7, 9].

## Conclusions

A first-trimester prognostic model for GDM was successfully implemented into obstetric care and was well received by OHP and pregnant women. We therefore recommended that prognostic models for GDM should be considered for adoption in obstetric guidelines. All areas using selective risk factor based testing for GDM could potentially benefit from using a prognostic model [38, 39]. Generalisability should be examined before implementing a prognostic model in a new population as model updating might be required to account for differences such as disease prevalence and predictor distribution [10, 40]. Furthermore, this study aggregated knowledge on how we could possible enhance our implementation strategy. The identified facilitators and barriers may as well provide insights into enhancing implementation of other (healthcare) innovations in various settings. Future studies are needed to broadly validate and improve quantitative implementation instruments in various healthcare fields. Additionally, normative data and directives for reporting are needed in order to classify what scores define a successful implementation and to make comparisons possible, serving both implementation research and practice.

## Supplementary Information

**Additional file 1: Table S1.** Full statements of the implementation survey administered to obstetric healthcare professionals to evaluate the implementation of a prognostic model for gestational diabetes mellitus including the NoMAD (a), MIDI (b), and extra evaluation (c). **Table S2.** Characteristics and outcomes of pregnant women stratified by responder type. **Table S3.** Median scores and internal consistency of MIDI and NoMAD instruments. **Table S4.** List of participating investigators of the RESPECT2 study. **Box S1.** Equation of the prognostic model for gestational diabetes mellitus. **Box S2.** Implementation strategies. **Box S3.** Diagnosis and treatment of gestational diabetes mellitus. **Box S4.** Definitions of characteristics and pregnancy outcomes. **Figure S1.** Receiver-operator-curve (a) and calibration plot (b) of the implemented prognostic model for gestational diabetes mellitus.

## Data Availability

The datasets analysed during the current study are available from the corresponding author on reasonable request.

## Abbreviations

BMI: Body mass index
GDM: Gestational diabetes mellitus
MIDI: Measurement Instrument for Determinants of Innovations instrument
NoMAD: Normalization MeAsure Development Instrument
OGTT: Oral glucose tolerance test
OHP: Obstetric healthcare professional(s)
RESPECT2: Risk EStimation for PrEgnancy Complications to provide Tailored care study part two
