# Supplementary material for: Implementation of a first-trimester prognostic model to improve screening for gestational diabetes mellitus
Source: BMC Pregnancy Childbirth. 2021 Apr 13;21:298. doi: 10.1186/s12884-021-03749-x (PMC8045273; doi:10.1186/s12884-021-03749-x)
Supplement: Supplementary file 1 — Additional file 1: Table S1. Full statements of the implementation survey administered to obstetric healthcare professionals to evaluate the implementation of a prognostic model for gestational diabetes mellitus including the NoMAD (a), MIDI (b), and extra evaluation (c). Table S2. Characteristics and outcomes of pregnant women stratified by responder type. Table S3. Median scores and internal consistency of MIDI and NoMAD instruments. Table S4. List of participating investigators of the RESPECT2 study. Box S1. Equation of the prognostic model for gestational diabetes mellitus. Box S2. Implementation strategies. Box S3. Diagnosis and treatment of gestational diabetes mellitus. Box S4. Definitions of characteristics and pregnancy outcomes. Figure S1. Receiver-operator-curve (a) and calibration plot (b) of the implemented prognostic model for gestational diabetes mellitus. [file 12884_2021_3749_MOESM1_ESM.docx]

Additional file 1. Supplemental tables, boxes and figures.

[**Table S1**.](#_Table_S1._Full) Full statements of the implementation survey administered to obstetric healthcare professionals to evaluate the implementation of a prognostic model for gestational diabetes mellitus including the NoMAD (a), MIDI (b), and extra evaluation (c).

[**Table S2**.](#_Table_S3._Characteristics) Characteristics and outcomes of pregnant women stratified by responder type.

[**Table S3**](#_Table_S4._Median). Median scores and internal consistency of MIDI and NoMAD instruments.

[**Table S4.**](#_Table_S5._Participating) List of participating investigators of the RESPECT2 study.

[**Box S1**.](#_Box_S1._Equation) Equation of the prognostic model for gestational diabetes mellitus.

[**Box S2**.](#_Box_S2._Implementation) Implementation strategies.

[**Box S3**.](#_Box_S3._Diagnosis) Diagnosis and treatment of gestational diabetes mellitus.

[**Box S4**.](#_Box_S4._Definitions) Definitions of characteristics and pregnancy outcomes.

[**Figure S1**.](#_Figure_S1._Receiver-operator-curve) Receiver-operator-curve (a) and calibration plot (b) of the implemented prognostic model for gestational diabetes mellitus.

[***References***](#_References) ***(online-only supplementary material)***

## Table S1. Full statements of the implementation survey administered to obstetric healthcare professionals to evaluate the implementation of a prognostic model for gestational diabetes mellitus including the NoMAD (a), MIDI (b), and extra evaluation (c).

| **(a)** | **NoMAD**^*^ |  |  |
| --- | --- | --- | --- |
| **No.** | **Subconstruct** | **Outcome** | **Item** |
| GN1^†^ | Past normality | appropriateness | When you use [the intervention], how familiar does it feel? |
| GN2^†^ | Current normality | feasibility | Do you feel [the intervention] is currently a normal part of your work? |
| GN3^†^ | Future normality | sustainability | Do you feel [the intervention] will become a normal part of your work? |
| C1 | Differentiation | acceptability | I can see how [the intervention] differs from usual ways of working |
| C2 | Communal specification | appropriateness | Staff in this organisation have a shared understanding of the purpose of [the intervention] |
| C3 | Individual specification | acceptability | I understand how [the intervention] affects the nature of my own work |
| C4 | Internalization | acceptability | I can see the potential value of [the intervention] for my work |
| CP1 | Initiation | feasibility | There are key people who drive [the intervention] forward and get others involved |
| CP2 | Legitimation | appropriateness | I believe that participating in [the intervention] is a legitimate part of my role |
| CP3 | Enrolment | adoption | I’m open to working with colleagues in new ways to use [the intervention] |
| CP4 | Activation | sustainability | I will continue to support [the intervention] |
| CA1 | Interactional workability | feasibility | I can easily integrate [the intervention] into my existing work |
| CA2^‡^ | Relational integration 1 | feasibility | [The intervention] disrupts working relationships |
| CA3 | Relational integration 2 | appropriateness | I have confidence in other people’s ability to use [the intervention] |
| CA4 | Skill set workability 1 | feasibility | Work is assigned to those with skills appropriate to [the intervention] |
| CA5 | Skill set workability 2 | feasibility | Sufficient training is provided to enable staff to implement [the intervention] |
| CA6 | Contextual integration 1 | feasibility | Sufficient resources are available to support [the intervention] |
| CA7 | Contextual integration 2 | feasibility | Management adequately supports [the intervention] |
| RM1 | Systemization | acceptability | I am aware of reports about the effects of [the intervention] |
| RM2 | Communal appraisal | acceptability | The staff agree that [the intervention] is worthwhile |
| RM3 | Individual appraisal | acceptability | I value the effects that [the intervention] has had on my work |
| RM4 | Reconfiguration 1 | sustainability | Feedback about [the intervention] can be used to improve it in the future |
| RM5 | Reconfiguration 2 | feasibility | I can modify how I work with [the intervention] |
| **(b)** | **MIDI** **^§^** |  |  |
| **No.** | **Subconstruct** | **Outcome** | **Item** |
| I1 | Procedural clarity | acceptability | [The innovation] clearly describes the activities I should perform and in which order. |
| I2 | Correctness | acceptability | [The innovation] is based on factually correct knowledge. |
| I4 | Complexity | acceptability | [The innovation] is too complex for me to use. |
| I5 | Compatibility | feasibility | [The innovation] is a good match for how I am used to working. |
| I6 | Observability | sustainability | The outcomes of using [the innovation] are clearly observable. |
| I7 | Relevance for client | appropriateness | I think [the innovation] is relevant for my clients. |
| U8 ^\|\|,¶^ | Personal benefit/drawback | acceptability | [The innovation] *improves the relationship with my client*/ *contributes to medicalisation^c^*. |
| U9 ^\|\|,¶^ | Outcome expectations | appropriateness | Pregnant women will become more aware of the influence of lifestyle on pregnancy complications. |
|  |  |  | Pregnant women with an increased risk for GDM will *consult a dietician*/ *eat more healthy*/ *perform more exercise*/ *accept screening for GDM.* |
| U10 | Professional obligation | appropriateness | I feel it is my responsibility as a professional to use [the innovation]. |
| U11 | Client satisfaction | appropriateness | Clients will generally be satisfied if I use [the innovation]. |
| U12 | Client cooperation | appropriateness | Clients will generally cooperate if I use [the innovation]. |
| U13 | Social support | feasibility | I can count on adequate assistance from my colleagues if I need it to use [the innovation]. |
| U14 | Descriptive norm | penetration | In your opinion, what proportion of the colleagues in your organization for whom [the innovation] is intended actually use the innovation? |
| U15^¶^ | Subjective norm:  normative beliefs | penetration | To what extent do(es) *your direct colleagues/ your local obstetric collaboration*/ *your* *regional obstetric collaboration/ pregnant women* expect you to use [the innovation]? |
|  | Subjective norm:  motivation to comply |  | When it comes to working in accordance with [the innovation], to what extent do you comply with the opinions of *your direct colleagues/ your local obstetric collaboration*/ *your* *regional obstetric collaboration/ pregnant women*? |
| U16^¶^ | Self-efficacy | feasibility | Should you wish to do so, do you think you can *apply the prognostic model for every pregnant woman*/ *discuss lifestyle advice with pregnant women with an increased risk for GDM*? |
| U17 | Knowledge | acceptability | I know enough to use [the innovation]. |

| O19^#^ | Formal ratification  by management | feasibility | Has the management set up formal arrangements in your organisation relating to the use of [the innovation] (in policy plans, work plans and so on)? |
| --- | --- | --- | --- |
| O20 | Replacement when  staff leave | sustainability | In my organisation, there are arrangements in place so that staff who use [the innovation] and leave the organisation are replaced in good time by employees who are adequately prepared to take over. |
| 021 | Staff capacity | feasibility | There are enough people in our organisation to use [the innovation] as intended. |
| O23 | Time available | feasibility | Our organisation provides me with enough time to include [the innovation] as intended in my day-to-day work. |
| O25 | Coordinator | feasibility | In my organisation, one or more people have been designated to coordinate the process of implementing [the innovation]. |
| O26^#^ | Unsettled organisation | feasibility | Are there, in addition to the implementation of [the innovation], any other changes in the organisation affecting the implementation of the innovation now or in the foreseeable future (reorganisation, merger, cuts, staffing changes, other innovations)? |
| O27 | Accessibility information | feasibility | It is easy for me to find information in my organisation about using [the innovation] as intended. |
| O28 | Performance feedback | sustainability | In my organisation, feedback is regularly provided about progress with the implementation of [the innovation]. |
| **(c)** | **Extra evaluation** |  |  |
| **No.** | **Subconstruct** | **Outcome** | **Item** |
| E1 | Continue model | Sustainability | I would like to continue using the prognostic model for GDM. |
| E2 | Continue care plan | Sustainability | I would like to continue using the care management plan with the prognostic model for GDM |
| E3 | New platform | Sustainability | I find the prognostic model on the new website and mobile application user friendly and I would be able to integrate it in my daily practice. |
| E4^**^ | Preferred platform | Sustainability | I would prefer to work with the prognostic model: 1) on the study website, 2) on the new website and mobile application, 3) integrated in the electronic patient file, 4) other, namely … |
| E5^††^ | Recommendations and  remarks | Sustainability | Do you have any recommendations or remarks about how you would like to work with the prognostic model for GDM in the future? |

Statements were measured on a 5-point Likert scale of 1 (strongly disagree) to 5 (strongly disagree). ^*^ The additional response option of the NoMAD instrument to indicate if a statement was applicable was not included. ^†^ Measured on a 0 (not at all) to 10 (completely) visual analogue scale. ^‡^ Conversed for analysis since this was a negative statement. ^§^ In accordance with MIDI regulations we made adjustments to ensure items were applicable and to avoid too many alike statements: MIDI item I3, O22 and O24 were replaced by the more general NOMAD item CA3 concerning the adequate availability of resources, MIDI item U10 was replaced by NOMAD item CP2 and MIDI item O25 was replaced by NoMAD item CP1; MIDI items I18 and O29 were not applicable. ^||^ Researchers had to state (U8) concrete expected benefits/drawbacks for the user and (U9) intended objectives of the innovation. ^¶^ all phrases in italic that are separated by a dash were questioned in separate statements and averaged to provide a single subconstruct score for analysis. ^#^ Dichotomous scale ('yes'/'no'). ^**^ Multiple choice. ^††^ Open-ended.

*No.*, abbreviated construct name plus item number. *NoMAD*, Normalization MeAsure Development instrument [1-3]. *GN,* global normalisation. *C,* coherence. *CP,* cognitive participation. *CA,* collective action. *RM,* reflexive monitoring. *MIDI*, Measurement Instrument for Determinants of Innovations [4]. *I,* innovation. *U,* user. *O,* organisation. *E,* extra evaluation. *[the innovation]*, the prognostic model and accompanying care map. *GDM*, gestational diabetes mellitus.

## Table S2. Characteristics and outcomes of pregnant women stratified by responder type.

|  | **Responder** | **Non-responder** |  |
| --- | --- | --- | --- |
|  | (n=672) | (n=401) | *p-value* |
| Age (years) | 31.7 (4.2) | 31.0 (4.5) | *0.015* |
| Body mass index (kg/m2) | 23.7 (21.5-26.9) | 24.1 (21.3-27.9) | *0.198* |
| Ethnicity (Caucasian) | 603 (89.7) | 301 (75.1) | *<0.001* |
| Parity (nulliparous) | 285 (42.4) | 155 (38.7) | *0.226* |
| Spontaneous conception | 624 (92.9) | 379 (94.5) | *0.288* |
| Pre-existent hypertension | 14 (2.1) | 4 (1.0) | *0.179* |
| Polycystic ovarian syndrome | 17 (2.5) | 7 (1.7) | *0.401* |
| History of gestational diabetes mellitus | 21 (3.1) | 10 (2.5) | *0.550* |
| History of macrosomia^*^ | 28 (7.3) | 11 (4.5) | *0.156* |
| History of unexplained intra-uterine fetal demise | 1 (0.1) | 3 (0.7) | *0.095* |
| Family history of diabetes mellitus^†^ | 106 (15.8) | 85 (21.2) | *0.025* |
| First trimester glucose (mmol/l) | 4.7 (4.4-5.1) | 4.8 (4.4-5.1) | *0.556* |
| High-risk for gestational diabetes mellitus^‡^ | 2018 (31.0) | 144 (35.9) | *0.094* |
| Gestational diabetes mellitus | 30 (7.5) | 51 (7.6) | *0.948* |
| Hypertensive disorders of pregnancy | 49 (12.2) | 85 (12.6) | *0.837* |
| Induction of birth | 100 (24.9) | 156 (23.2) | *0.743* |
| Mode of birth (spontaneous) | 309 (82.6) | 531 (85.5) | *0.597* |
| Postpartum hemorrhage >1000 ml | 32 (8.0) | 55 (8.2) | *0.900* |
| Maternal death | 0 (0) | 0 (0) | *NA* |
| Gestational age at birth (days) | 279 (272-286) | 281 (273-286) | *0.175* |
| Birthweight (grams) | 3470 (3079-3791) | 3520 (3174-3866) | *0.027* |
| Small-for-gestational-age^§^ | 58 (14.5) | 66 (9.9) | *0.0214* |
| Large-for-gestational-age^*^ | 44 (11.0) | 74 (11.0) | *0.984* |
| Apgar score <7 after 5 minutes | 7 (1.8) | 12 (1.8) | *0.966* |
| Shoulder dystocia | 11 (2.7) | 19 (2.8) | *0.932* |
| Birth trauma | 3 (0.8) | 3 (0.4) | *0.518* |
| Hypoglycemia <2.6 mmol/L | 40 (10.0) | 67 (10.0) | *0.930* |
| Neonatal intensive care unit admission | 12 (3.0) | 19 (2.8) | *0.972* |
| Perinatal death >22 weeks gestational age | 1 (0.2) | 1 (0.1) | *0.709* |

Values are mean (SD), median (interquartile range) or number (percentage). ^*^ Birthweight percentile >90 [5]. ^†^ First degree family member with any type of diabetes mellitus. ^‡^ High-risk for GDM according to the implemented prognostic model (predictors in prognostic model: age, body mass index, ethnicity, first trimester glucose level, family history of diabetes, GDM in a previous pregnancy). ^§^ Birthweight percentile <10 [5]. *GDM,* gestational diabetes mellitus. *Low risk,* low risk for GDM. *High risk,* high risk for GDM. A pregnant woman was defined as a responder when she filled out one or both questionnaire(s).

## Table S3. Median scores and internal consistency of NoMAD and MIDI instruments.

|  | (n=42) |  |  |
| --- | --- | --- | --- |
|  | *Median (IQR)* | α | α – 1 |
| NoMAD and MIDI combined | 3.7 (3.3-4.0) | 0.92 | - ^*^ |
| NoMAD | 3.7 (3.3-3.9) | 0.88 | - ^*^ |
| Coherence | 4.0 (3.5-4.0) | 0.72 | 0.78 (C2) |
| Cognitive participation | 3.9 (3.3-4.3) | 0.73 | 0.82 (CP1) |
| Collective action | 3.4 (3.0-3.7) | 0.66 | 0.67 (CA3) |
| Reflexive monitoring | 3.6 (3.2-3.8) | 0.65 | 0.68 (RM1) |
| MIDI | 3.6 (3.1-3.8) | 0.91 | - ^*^ |
| Innovation | 3.6 (3.1-4.0) | 0.75 | 0.76 (I4) |
| User | 3.7 (3.4-3.9) | 0.78 | 0.80 (U14) |
| Organisation | 3.2 (2.7-3.6) | 0.80 | 0.83 (O19) |
| NoMAD global normalization items |  |  |  |
| Past normality | 7.5 (6.5-8.1) | NA | NA |
| Current normality | 7.0 (5.1-8.2) | NA | NA |
| Future normality | 8.0 (7.0-9.0) | NA | NA |

NoMAD and MIDI subconstructs were scored on a 5-point Likert scale from 0 (strongly disagree) to 5 (strongly agree). NoMAD global normalization items were scored on a visual analogue scale from 0 (not at all) to 10 (completely). ^*^ no improvement. α*,* Cronbach’s Alpha. *α – 1,* best possible Cronbach’s Alpha when one subconstruct is dropped (item code of deleted subconstruct). *NoMAD*, Normalization MeAsure Development instrument [1-3]. *MIDI*, Measurement Instrument for Determinants of Innovations [4].

## Table S4. Participating investigators of the RESPECT2 study.

| **Name** | **Affiliation** | **Obstetric collaboration organization** |
| --- | --- | --- |
| Anjoke J.M. Huisjes | Gelre Hospitals, Apeldoorn, the Netherlands | Apeldoorn ‘Verloskundig Samenwerkingsverband Apeldoorn’  Hospital: Gelre Ziekenhuizen  Midwifery practice: Ezra |
| Eva Stekkinger | Deventer Hospital, Deventer, the Netherlands | Deventer ‘Geboortezorg Salland’  Hospital: Deventer Ziekenhuis  Midwifery practices: Anno, Baren en Zo, De Kuip, de Eiber. |
| Mireille N. Bekker | University Medical Center Utrecht, Utrecht, the Netherlands | Utrecht ‘Alliant Geboortezorg’  Hospital: University Medical Centre Utrecht  Midwifery practices: Universitair Verloskundig Centrum, Het Wonder, Maarsenbroek, Zeist. |

## Box S1. Equation of the prognostic model for gestational diabetes mellitus.

$Probability to develop GDM= \frac{e^{x}}{1+e^{x}}$

x = -10.6121880 + 0.608744 if age 25-19 years + 0.787984 if age is 30-34 years + 1.068414 if age 35-39 years + 1.648934 if older over 39 years + 0.839521 if BMI 20.0–24.9 + 1.535419 if BMI 25.0-26.9 + 1.664914 if BMI 27.0-29.9 + 2.399441 if BMI 30.0-34.9 + 2.568771 if BMI over 34.9 + 0.93596 if Asian + -0.844531 if African + 1.065957 if first degree relative with diabetes mellitus + 2.039894 if history of GDM + 3.233494 * first trimester venous glucose level.

The model was originally developed by Teede et al 2011 and externally validated and updated by our group [6, 7]. Among consensus within the study group, a predicted probability threshold was chosen where as many women as compared to the previous selective screening approach^*^ would be tested aiming to identify more GDM cases. The coefficients of the model were refitted on the validation cohort [7] and the threshold for high-risk was calculated to be >4.6% predicted risk on GDM.

*^*^ High-risk for GDM: BMI >30 kg/m2, previous macrosomia, history of GDM, family history of diabetes, non-western ethnicity, polycystic ovarium syndrome, or previous unexplained fetal demise [8].*

## Box S2. Implementation strategies.

One key person per site was assigned to be concerned with the implementation of the prognostic model to build a coalition and for train-the-trainer purposes. In educational outreach visits, healthcare professionals received (individual) training on site. Before focusing on how to practically use the prognostic model, the dynamic training sessions started with theoretical background including the expected change. Extra training sessions were performed on request, and offered actively to centres where the model was used far less than expected. Educational material was developed and distributed, including a manual on how to apply the model and care pathways and contact details to centralize technical assistance. Monthly newsletters were disseminated in order to share local knowledge, promote adaptability and conduct ongoing training by sharing user tips, reward early adopters and champions, and to remind OHCP.

## Box S3. Diagnosis and treatment of gestational diabetes mellitus.

GDM was diagnosed with a 2-hour 75-grams OGTT. Due to a transition in diagnostic thresholds, two hospitals and five midwifery practices used the WHO 1999 criteria (venous plasma glucose fasting >7.0 mmol/L or 2-hour post load >7.8 mmol/L) and one hospital and four midwifery practices used the WHO 2013 criteria (venous plasma glucose fasting >5.1 mmol/L, 1-hour post load >10.0 or 2-hour post load >8.5 mmol/L) to diagnose GDM. Analyses were performed on the clinical diagnosis of GDM unless stated otherwise. Women with GDM started treatment with antidiabetic medication when glycaemic control was not achieved with dietary and lifestyle counselling by a dietician or diabetes specialist nurse; glucose >5.3 mmol/L fasting, >7.8 mmol/L 1-hour postprandial or >6.7 mmol/L 2-hour postprandial.

## Box S4. Definitions of characteristics and pregnancy outcomes.

Characteristics of pregnant women

Age (years), height (centimetres), weight (kilograms), ethnicity (Caucasian, North African, Turkish, Asian, Sub-Saharan African, Hindustani, Latin American, other or mixed), parity, method of conception (spontaneous/assisted), estimated date of delivery, first degree family member with any type of diabetes, first trimester venous glucose, medical history (hypertension, kidney disease, thyroid disorder, cardiovascular disease, polycystic ovarium syndrome, thromboembolic event, psychiatric disorder), obstetric history (gestational diabetes mellitus, large-for-gestational-age neonate (>90^th^ birthweight percentile), preterm birth (<37 weeks of gestation), preeclampsia, caesarean section, postpartum haemorrhage (>1000ml), intra-uterine fetal demise (explained/unexplained). Body mass index (BMI) was calculated by dividing weight in kilograms by squared height in meters.

Pregnancy outcomes

Clinical diagnosis of preeclampsia [9], pregnancy induced hypertension [9], thromboembolic event, onset of birth (spontaneous, induction, primary caesarean section), mode of birth (spontaneous, assisted vaginal delivery, secondary caesarean section), perineal tear grade 3 or 4, postpartum haemorrhage (>1000ml) with or without blood transfusion, maternal death. Neonatal outcomes include sex, gestational age at birth (days), preterm birth (<37 weeks of gestation), birthweight (grams), small- or large-for-gestational-age neonates (<10^th^ or >90^th^ birthweight percentile [5]), Apgar-score <7 after 5 minutes, shoulder dystocia, birth trauma, glucose measurement, hypoglycaemia (mild <2.6 mmol/l; severe <2.0 mmol/l; requiring intravenous glucose treatment), hyperbilirubinemia, respiratory disorder, congenital anomaly, neonatal intensive care unit (NICU) admission, perinatal death (from 22 weeks of gestation until 6 weeks postpartum), termination of pregnancy (<24 weeks of gestation).


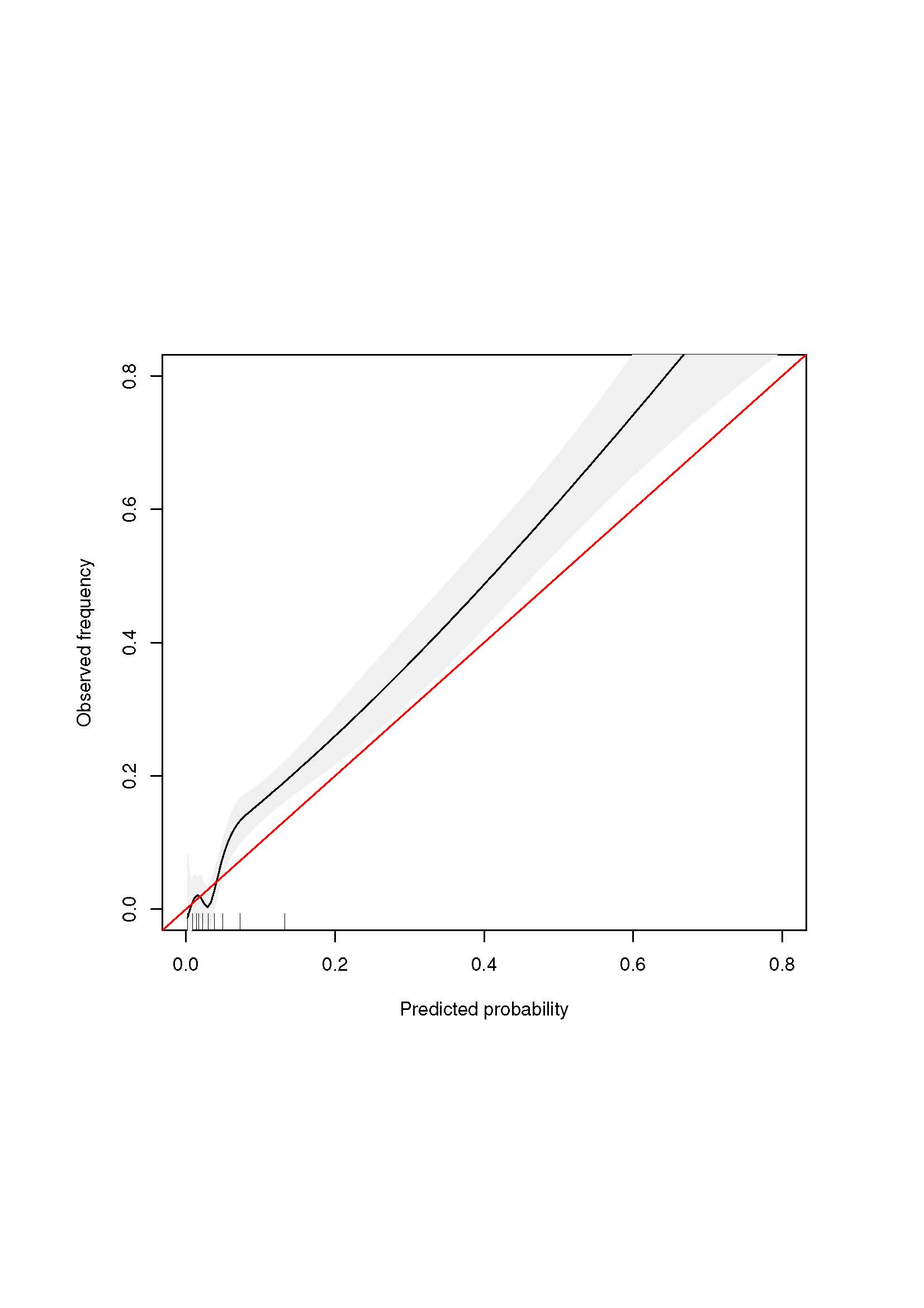

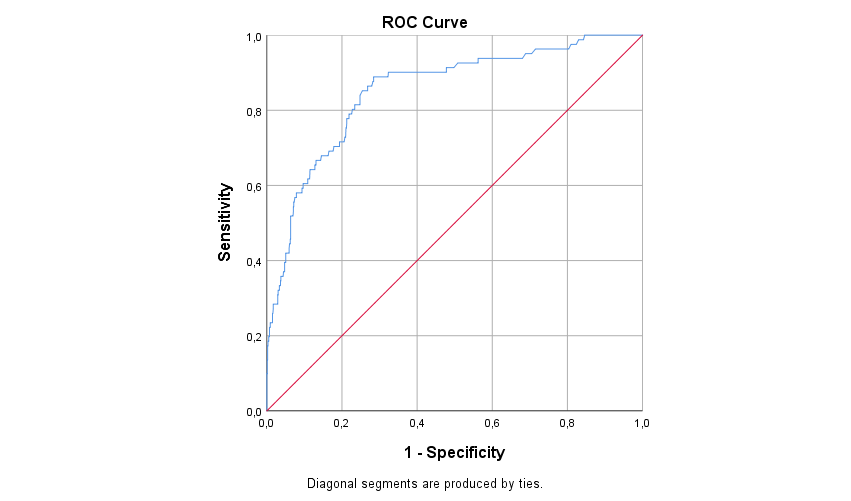
**(a) (b)**

**Observed frequency**

**sensitivity**

c-statistic 0.85 (95%CI 0.81-0.90)

**predicted probability**

**1-specificity**

## Figure S1. Receiver-operator-curve (a) and calibration plot (b) of the implemented prognostic model for gestational diabetes mellitus.

## References

1. Finch TL, Girling M, May CR, Mair FS, Murray E, Treweek S, et al. Improving the normalization of complex interventions: part 2 - validation of the NoMAD instrument for assessing implementation work based on normalization process theory (NPT). BMC Med Res Methodol. 2018;18(1):135.

2. Rapley T, Girling M, Mair FS, Murray E, Treweek S, McColl E, et al. Improving the normalization of complex interventions: part 1 - development of the NoMAD instrument for assessing implementation work based on normalization process theory (NPT). BMC Med Res Methodol. 2018;18(1):133.

3. Vis C, Ruwaard J, Finch T, Rapley T, de Beurs D, van Stel H, et al. Toward an Objective Assessment of Implementation Processes for Innovations in Health Care: Psychometric Evaluation of the Normalization Measure Development (NoMAD) Questionnaire Among Mental Health Care Professionals. J Med Internet Res. 2019;21(2):e12376.

4. Fleuren MA, Paulussen TG, Van Dommelen P, Van Buuren S. Towards a measurement instrument for determinants of innovations. Int J Qual Health Care. 2014;26(5):501-10.

5. Hoftiezer L, Hof MHP, Dijs-Elsinga J, Hogeveen M, Hukkelhoven C, van Lingen RA. From population reference to national standard: new and improved birthweight charts. Am J Obstet Gynecol. 2019;220(4):383 e1- e17.

6. Teede HJ, Harrison CL, Teh WT, Paul E, Allan CA. Gestational diabetes: development of an early risk prediction tool to facilitate opportunities for prevention. Aust N Z J Obstet Gynaecol. 2011;51(6):499-504.

7. Lamain-de Ruiter M, Kwee A, Naaktgeboren CA, de Groot I, Evers IM, Groenendaal F, et al. External validation of prognostic models to predict risk of gestational diabetes mellitus in one Dutch cohort: prospective multicentre cohort study. BMJ. 2016;354:i4338.

8. Nederlandse Vereniging voor Obstetrie & Gynaecologie. Diabetes mellitus en zwangerschap [Guideline]. 2010 [updated 04-06-2010. Available from: <https://www.nvog.nl/wp-content/uploads/2018/02/Diabetes-mellitus-en-zwangerschap-2.0-04-06-2010.pdf>.

9. Brown MA, Magee LA, Kenny LC, Karumanchi SA, McCarthy FP, Saito S, et al. Hypertensive Disorders of Pregnancy: ISSHP Classification, Diagnosis, and Management Recommendations for International Practice. Hypertension. 2018;72(1):24-43.
